# Supplementary material for: Sinorhizobium meliloti GR4 Produces Chromosomal- and pSymA-Encoded Type IVc Pili That Influence the Interaction with Alfalfa Plants
Source: Plants (Basel). 2024 Feb 25;13(5):628. doi: 10.3390/plants13050628 (PMC10934763; doi:10.3390/plants13050628)
Supplement: Supplementary file 1 [file plants-13-00628-s001.zip › plants-2841360-supplementary.pdf]

Supplementary Materials

Sinorhizobium meliloti GR4 produces chromosomal- and pSymA-encoded Type IVc Pili that influence the interaction with alfalfa plants

Cristina Carvia-Hermoso[1], Virginia Cuéllar[1], Lydia M. Bernabéu-Roda[1], Pieter van Dillewijn[1,*], María José Soto[1,*]

List of Supplementary Material

Figure S1. Predictive protein composition and structure of the secretion systems encoded by *S. meliloti tad/cpa* genes of the *flp-1* cluster, *flp-2* cluster and Locus 3.

Figure S2. Multiple sequence alignment by MUSCLE of Flp prepilins.

Figure S3. Multiple sequence alignment by MUSCLE of PilQ2-like gene products.

Figure S4. Multiple sequence alignment by MUSCLE of SMa0164 with homologous proteins of *S. fredii* NGR234 (Sf) and *B. diazoefficiens* (Bd).

Figure S5. Multiple sequence alignment by MUSCLE of SMa0166 with TadD-like gene products.

Figure S6. Transmission electron microscope (TEM) images of flagella-plus single and double *flp* deletion mutants.

Figure S7. Twitching motility assay of duplicate stab inoculations of the different strains on different media with different agar concentrations and types.

Figure S8. Appearance of alfalfa plants inoculated with *S. meliloti flp* mutant strains at the end of the nodulation kinetics experiment.

Table S1. Percent sequence identities (in parenthesis) among *S. meliloti* T4cP paralogues.

Table S2. Strains and plasmids used in this study.

Table S3. List of primers used in this study.

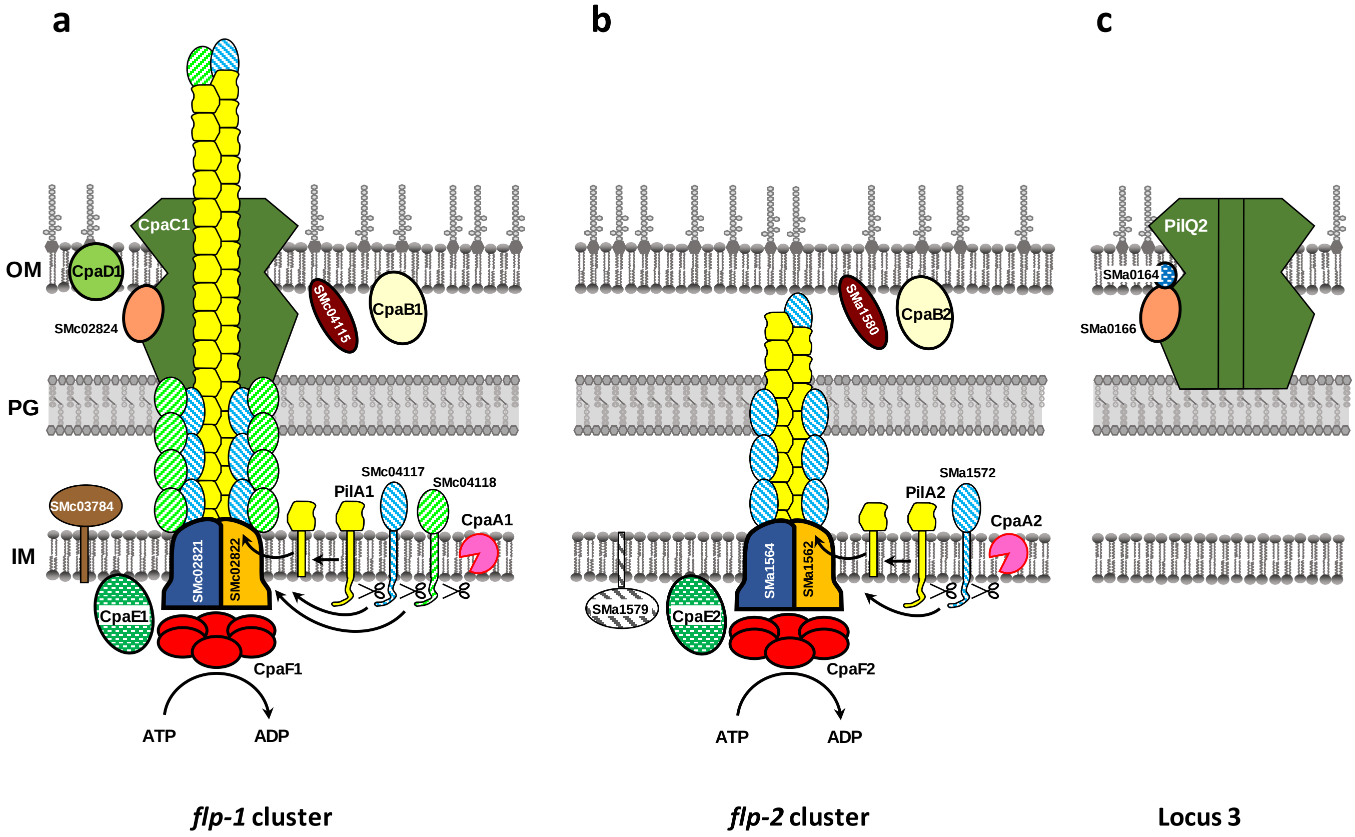

Figure S1. Predictive protein composition and structure of the secretion systems encoded by *S. meliloti tad/cpa* genes of the *flp-1* cluster (**a**), *flp-2* cluster (**b**) and Locus 3 (**c**). See text in the main manuscript for details.

a

```
Sm SMc02446    -METLRRLLRDHDGATAVEYGLLAALISVGLLIGLQNFSGALLGMLTFVTNTIEAAWI--
Sm PilA1       MKTIFARLMKDESGATAIEYGLIAALISVALIGGAQTLGGALSTQFTNLGGYLNVEPNAP
Sm PilA2       MKNLLARFARNESGATAIEYGLIAGLISVVLITVMGTIGTGLTTRFTAIGTALTGG----
                   : *: .: .****:****:*.**** *:    .:. .*   :* :   :
```

b

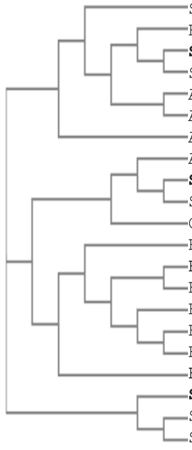

```
Sf NGR_b10880            ------------------------------------------------MKAFVTKVSAFAREEDGVALSEYLVLLGLL--VGGVIAAV---TTVSGN-LAAAWTSWGTFFTSHLSCDPAAPLC
Bd PilA                  MLRITPLWSTRNPPSEHYKRIQLAQGFFANEHSEVCHLQGRDPPMLTYYIETREALKRLRTDQDGVVSFEYIIVAVCIVGAVGAVFGGGAGGQIGAA-LTTGITAITTAFATAIAG-------
Sm SMc02446              ----------------------------------------------------METLRRLLRDHDGATAVEYGLLAALI--SVGLLIGL---QNFSGA-LLGMLTFVTNTIEAAWI--------
Sf fig|394.7.peg.5489    ----------------------------------------------------MDKLRSLVRNRDGATIVEYGLLAALI--SVALLVGL---QGFSNE-LLNVLNLIADAIEDSWT--------
Aa Flp-2                 ----------------------------------------MMDLLDYFYRQVVFFSCNFYRNRQGITSVEYGLIAVAI--AVFVVAVLVGDNSFIKA-LSGKFSDLTTTVSGAIVSKSS----
Aa Flp-1                 ---------------------------------------MLNTLTTKAYIKASEAIRSFRENQAGVTAIEYGLIAIAV--AVLIVAVFYSNNGFIAN-LQSKFNSLASTVASANVTK------
Af PilA                  ------------------------------------MPVVETHRMVDGEYPVLHFFINFCKNENGATAIEYGLIAGII--SAALIAGL---GNISSG-INAVFQFIVDAFPKG----------
Af CtpA                  ---------------------------------------------------MTKIFARFLKDESGATAIEYGLIAALI--SVAIIGGA---STLGGK-LKDTFTFIGKSFTDSKASTGGV---
Sm PilA1                 ---------------------------------------------------MKTIFARLMKDESGATAIEYGLIAALI--SVALIGGA---QTLGGA-LSTQFTNLGGYLNVEPNAP------
Sf NGR_c34640            ---------------------------------------------------MKTIFARLMKDESGATAIEYGLIAALI--SVALITGA---TALGDS-LDSMFNALSGQMTTAETNM------
Cv PilA                  ---------------------------------------------------MTKFVTRFLKDESGATAIEYGLIVALI--AVVIVTAV---TTLGTN-LRTAFTKAGAAVSTAAGT-------
Bd Bsl3118               ----------------------------------------------------MALLKSFLADESAATAIEYCLIAAGI--ALVIVTVV---NNTGSA-LLNNKFNSIDAATK-----------
Bd Bsr1550               ----------------------------------------------------MKTLVHFLRDESGATAIEYGLIAAGI--SLAIIAVV---NGLGTK-LNTKFASISSSLK------------
Bd Bsl4812               ------------------------------------MICEHACQRYREGATMKNTLKNFLADERGATAIEYGLIAAGI--ALAVITVV---NGMGSK-LNTKFGSISTSLK------------
Bd Bsl6587               ---------------------------------------------------MTRMILKFWSNESGATAIEYGLIAAGI--ALAIITVV---NGLGTT-MNEKFTSISTSLK------------
Bd Flp-1                 ---------------------------------------------------MKNLIARFAKDESGATAIEYGLIAAGI--ALAIITVV---NNLGST-LNTKFTSISTSLK------------
Bd Bsl7141 Locus3        ---------------------------MVGRRWEAEETRPVVASCTFWEHSMKNLVARFVKDESGATAIEYGLIAAGI--ALAIITVV---NNLGST-LNTKFTSISTSLK------------
Bd fig|224911.5.peg.4259 -------------------------------------------------MKLKCLLADFAADESGATAIEYGLIAAGI--ALAIIEII---YALGTN-LVAKLQSLATALK------------
Sm PilA2                 ---------------------------------------------------MKNLLARFARNESGATAIEYGLIAGLI--SVVLITVM---GTIGTG-LTTRFTAIGTALTGG----------
Sf NGR_b08110            ---------------------------------------------------MKNLLLRFVRHESGATAIEYGLITGLI--AV--ITAV---QTVGTD-IDAAFDAIAAAL-------------
Sf NGR_b03720            ---------------------------------------------------MKNLLVRFVRNESGATAIEYGLIAGLI--AVVIISAV---QLVGTD-IGAKFTAISTAL-------------
                                                                                    :     . .  ** ::   :      :             :
```

Figure S2. Multiple sequence alignment by MUSCLE of Flp prepilins. (**a**) Alignment of the three putative Flp prepilins of *Sinorhizobium meliloti* (Sm). (**b**) Alignment of the Flp prepilins of *S. meliloti* with known Flp/PilA/CtpA prepilins of *Aggregatibacter actinomycetemcomitans* (Aa), *Caulobacter vibrioides* (Cv), *Agrobacterium fabrum* (Af), putative prepilins of *Sinorhizobium fredii* NGR234 (Sf), and *Bradyrhizobium diazoefficiens* (Bd). Identical residues are indicated with an asterisk. Colons and full stops indicate partial matches. The Flp motif is highlighted in grey. In (**b**) the cladogram obtained in MUSCLE is also shown.

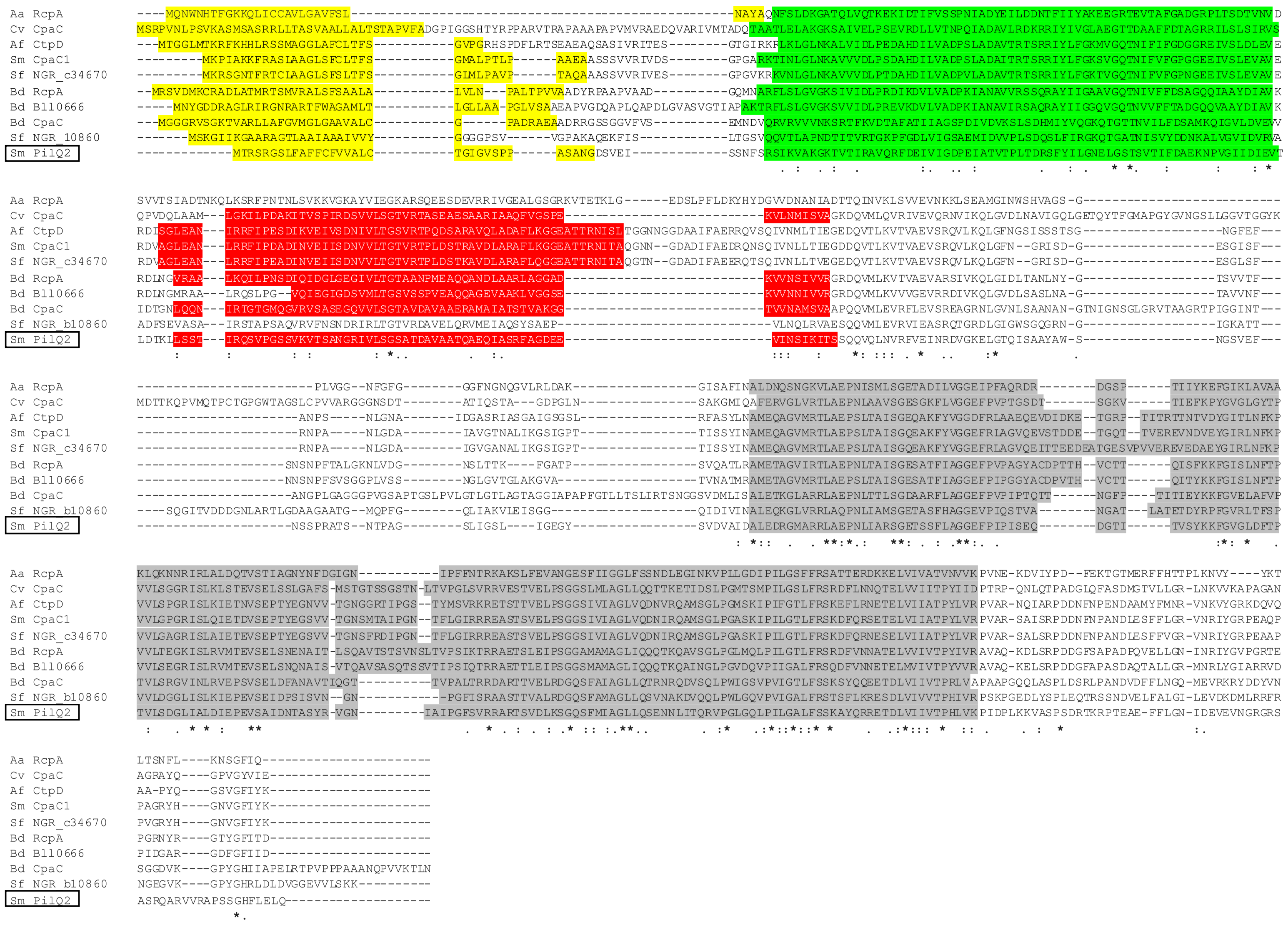
Aa RcpA
Cv CpaC
Af CtpD
Sm CpaC1
Sf NGR_c34670
Bd RcpA
Bd Bll0666
Bd CpaC
Sf NGR_b10860
Sm PilQ2

Figure S3. Multiple sequence alignment by MUSCLE of PilQ2-like gene products. Alignment of the amino acid sequences of PilQ2 (SMa0163 Locus 3) of *S. meliloti* with PilQ2-like gene products of *A. actinomycetemcomitans* (Aa), *C. vibrioides* (Cv), *A. fabrum* (Af), *S. fredii* NGR234 (Sf) and *B. diazoefficiens* (Bd). Identical residues are indicated with an asterisk. Colons and full stops indicate partial matches. The Signal peptide motif (Phobius) is highlighted in yellow, the T2SS-T3SS pil N: Pilus formation protein, N-terminal domain (InterPro IPR032789) in green, the BON domain (Pfam PF04975) in red and the T2SS/T3SS Dom: Type II/III secretion system, secretin-like domain (IPR004846 InterPro) in grey.

```
Sf NGR b10850     MSFPRSKRAI----LVLTAAGILLSGC---------ADYLNHRDSITFGLGNAVEANKGIH
Sm SMa0164        -MIRRSSRPRGFPGLLFASSIILLSGC--------QNHELVRSETIALSAGDAIAANSVMQ
Bd blr3501        -MTKRY--------LVLFAPLILLGGCYGLAGNDEMDRYFQRSNTITMSAGDAKEVNAVTH
                      *         *:: :. **.**               : . ::*::. *:*  .*   :
Sf NGR b10850     IQDPFPRAAYNTQIASDGKVIHRAIRTYQGSAQTSAP-----------------------
Sm SMa0164        MVDPWPPRVKQTSLATPAD-----LEQYKPQQPNAEQ-----------------------
Bd blr3501        TIHPWPRNVGDRRIAYDARRVGAAVTRYGKTQQPVDQLPDISDPTRQMGVRTTPTQNVNI
                     *:*  . :  :*  .       :  *
Sf NGR b10850     ------SPSAVILPVATPPNGAAR
Sm SMa0164        -----NGGNGETYPNDTTTQ----
Bd blr3501        EGLGAGSSTGVSVPVGGTGGAPNR
                        . ..   *   .
```

Figure S4. Multiple sequence alignment by MUSCLE of SMa0164 with homologous proteins of *S. fredii* NGR234 (Sf) and *B. diazoefficiens* (Bd). Identical residues are indicated with an asterisk. Colons and full stops indicate partial matches. The prokaryotic membrane lipoprotein lipid attachment site profile is highlighted in yellow. The lipobox motif (LXXC) is indicated with a box.

```
Aa TadD          ----------MYSKSLKNVLLCGMILSVTACSAVLNKKPLSAD--------KLAAKEALYQSTNNNDALITMYRDGLKNKEDPLTR---YKLSEIYYKKGDSNSSLLYLQPLL----TNS
Bd TadD Locus2   MSPMSRAGTTVMRRIIILMLIYCCLGTGLAGCDYTLREAAIVAP----------------------VDPPGG----------DPVQEPTDVKY----------------------YPSDE
Sf NGR_b10740    ------MIDQIQSRLGRLSPLLLAALVLSGCQSAAFDDVAAFG-----------------DSAKTLEDDSAVAFY-------------------------------------------KN
Sm SMa0166       ------------MEMTTGASGRSV---LGRSGGGRRPTELA----------MAIVATVILSSCQTSEVLSGAEF-------DPTSALASSGDVS--------------------------
Cv CpaO          ---------MCRKRALIATVLAPIALVLATPVFAADPPKADAGKTAKAAPAPVKASAQQRTEARRLDPLAQAAFWGAEFEVDPADAEAGAGLAKALRALGRADEAAIAATKGLIAHPDDT
Af Atu0215       -MTAFSLGETRRTGLLRGACLAALVLAVSGCASTNKPDRMSTG--------SIPAATRSYDHLNMEQLRQAEETAGKAYERNPKDRSVGMNYASVLMMTGKNTQALAVMQQIAIANPADR
Sm SMc02824      -MTMQDNFSFSLRRLATGVAVAMVALSLSACAGQQGRKELTTG--------SIPKLTRPVQSMNATELAAAAERIGQAYERNPKDREAGLNYANLLRMTGRNEQALAVMQQVAIYHPADR
Sf NGR_c34740    -----------MHRLAVGAAVALMALALGGCAGQQGKKELTTG--------SIPKLSKPVQSMNATELAAAAENIGQAYERDPKNREAGLNYANLLRMSGRNEQALAVMQQVAINHPTDR
Bd TadD Locus1   ----MRQRFSPARLLASASLVAVVAMSLGGCTAMSKLSDV-TG--------SIGSRA---EASPPSDPAGAVEVYGERYRANPKDAEAALGYGQALRANGQRAQAAAVLEQATIANPGNK
Bd Blr0663       ----MSKRLSLASRPARYLLSALVLLALGGCQT-TGIEDI-TG--------ALGGKS---EAASKADGKPDMDALRERYRAKPSDPNVALDYGKALRESGQSAQAVAVLEQAVLGHPRNK
                                            :                                      :

Aa TadD          GQLSEKAKVLQARNLNQLKRYQEALEVENSLLTTSPKNGEVYNLRGVTYALMGNPNKANEDINKAREYFLNDAVAVNNLAMLSIVNGDYRNAVSLLLPQYLNGVREQRLVHNLIFALIKN
Bd TadD Locus2   PV--RLGLEHFNRG-----SYGLSQRYFKDAVEKSPKDLTAWIGLAASYDRLRRFDLADQAYAQAIRLGGETVQILNDQGYSYMLRGNLSAARRKFEKAYSLDPGNPVIANNLELLNGS-
Sf NGR_b10740    DELITTGKLQFKEK-----NYGKSYAIYKRAVAVFPQDPSAWLGFAASADMIGRFDTSDRAYQQLSKMIGNSPVYYNNMGYSRLLRGDLPRARQYFLKAYELDPANETTARNLEIM----
Sm SMa0166       KSDLDQGKLQFMNG-----NYGLAEKHFRKAVELRQDNAEALMGLAACYDRLGRFDLADRAYNQLLKVAGRQPRIVNNMGYSQYLRGEKAKARKLLLEARAASPGDETIEANLALL----
Cv CpaO          GLLLELARAHIARG-----QGFYAIEPARKVAALSPKDWRPLTLLGVAYEQAERDEEAEAAHRQAIALAPNEATPMANHAMHLAARGDLAGAEAELRRAVTLPSAGIQVRQNLALVVGLQ
Af Atu0215       EVLAAYGKSQAAAG-----QLEQALSTIQRAQTPDRPDWRLYSAEGAVLDQLGRSNEARSRYRQALDLKPNDPSVLSNLGMSYVLSSDPRTAETYLQSAISQPGADSRVRQNLALVVGLQ
Sm SMc02824      EVLGAYGKAQAAAG-----QLEQALATISRAQTPDRPDWKLKSAEGAILDQLGRSAEARLRYREALDLKPNEPSVLSNLGMSYLLTKDLRTAETYLKSAASQPDAGSSVRQNLALAVGLQ
Sf NGR_c34740    EVLGAYGKAQAAAG-----QLEQALQTIGRAQTPDRPDWKLKSAEGAVLDQLGRSSDARLRYREALDLQPNEPTILSNLGMSYLLTKDLKTAETYLRSAAEQPGADSSVRQNLALAVGLQ
Bd TadD Locus1   ALLALYGRALADNG-----NFQQAFDVLSKAHSPDNPDWRLLSVQGTALDQMGRHDEARSYYASALKIAPGDPGVLSNLGLSYMLSRDLPKAEEALRQAYASPRASARVRQNLGLVVGLQ
Bd Blr0663       ALLAGYGRALADNG-----NFQQAFDVLSRAHTPEDPDWRILSAQGAALDQLGRNEEAQQYYATALKIVPDEPQVLSNLGLSYVLQNNLPKAEQVLGRAHQRNQNDARIRANLALVLGLQ
                       .                :             :       ..      .   :                   : .       :   *   :               ** :

Aa TadD          NDIDYAKDIIVKENINTSPDDLINALKKTERVSNNIAR--------------
Bd TadD Locus2   --------------------------RRFIERPPNNQP--------------
Sf NGR_b10740    -------------------KNSVKYAQR------------------------
Sm SMa0166       -----------------------------------DRS--------------
Cv CpaO          GRLPEAEKLVRADLPPEQVANNLAYLRAAVGQAGQSRSWDAMRAGGGR----
Af Atu0215       GRFAEAERIAVQELSAQQAQANLTYLRAMLSQQNSWQQLAKKDRKPAG----
Sm SMc02824      GRFQEAETIAGQELTSEQAEANVAYLRSVLSQQGAWKQLAKAD---------
Sf NGR_c34740    GRFQEAETIARQELTPEQAEANVAYLRSMLSQKGAWTELAKADDGNTN----
Bd TadD Locus1   GRFAEAETIVKADLPPEQAAANVAYLKEMLSRSDAPRGAPKRTPVAALSRPD
Bd Blr0663       GREAEAEILVKADLPPDQAAAKITALRQLLAKKQQRAEK-------------
```

Figure S5. Multiple sequence alignment by MUSCLE of SMa0166 with TadD-like gene products. Alignment of the amino acid sequences of SMa0166 (Locus 3) of *S. meliloti* with TadD-like gene products of *A. actinomycetemcomitans* (Aa), *C. vibrioides* (Cv), *A. fabrum* (Af), *S. fredii* NGR234 (Sf) and *B. diazoefficiens* (Bd). Identical residues are indicated with an asterisk. Colons and full stops indicate partial matches. The Signal peptide motif (Phobius) is highlighted in yellow and the Tetrapeptide-like helical domain superfamily (TPR-motif) (InterPro IPR011990) is highlighted in grey. The prokaryotic membrane lipoprotein lipid attachment site profile (PROSITE PS51257) and the lipobox motif in SMa0166 are indicated in red letters.

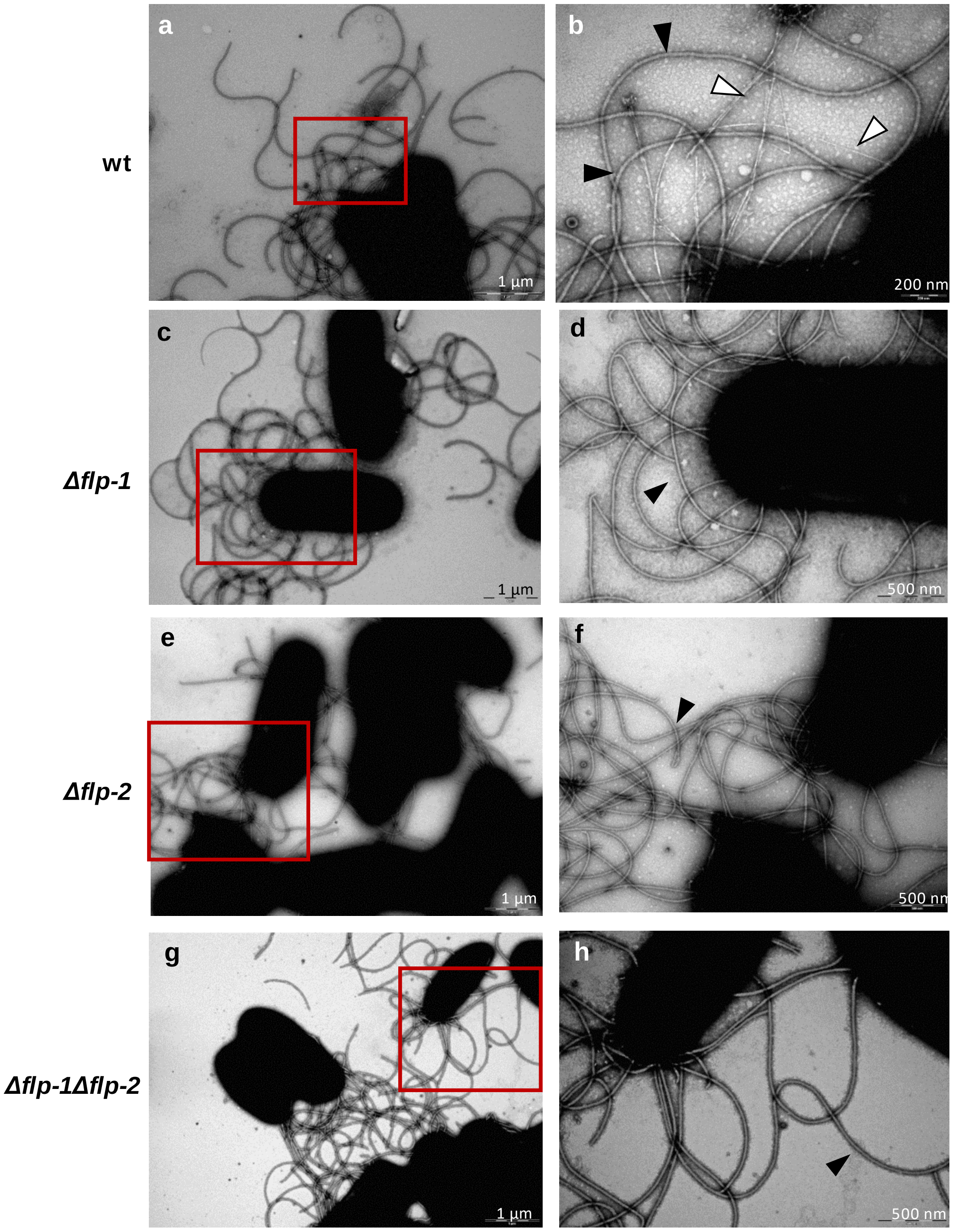

Figure S6. Transmission electron microscope (TEM) images of flagella-plus single and double *flp* deletion mutants. Cells were grown on the surface of Minimal Medium (1%) and negatively stained with uranyl acetate (2%). Red squares indicate the zone shown at higher magnification on the right. Solid and open arrowheads indicate flagella and pili-like structures, respectively.

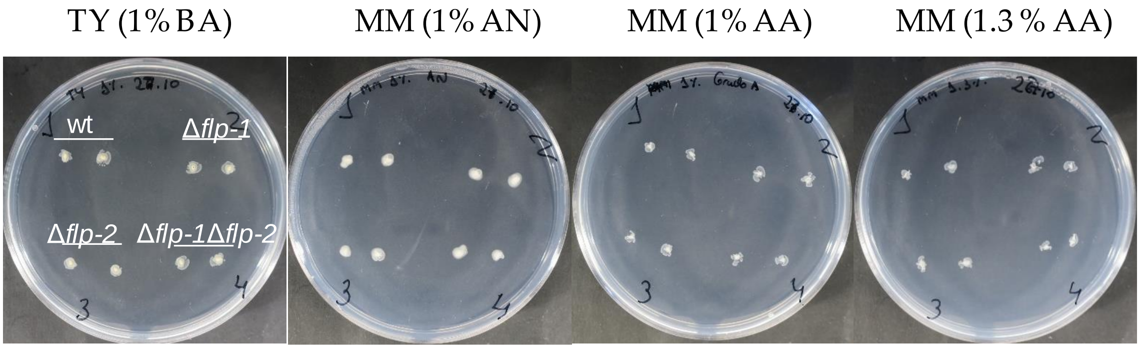

Figure S7. Twitching motility assay of duplicate stab inoculations of the different strains on different media with different agar concentrations and types. BA: Bacteriologic Agar; AN: Agar, Noble; AA: Agar, Grade A.

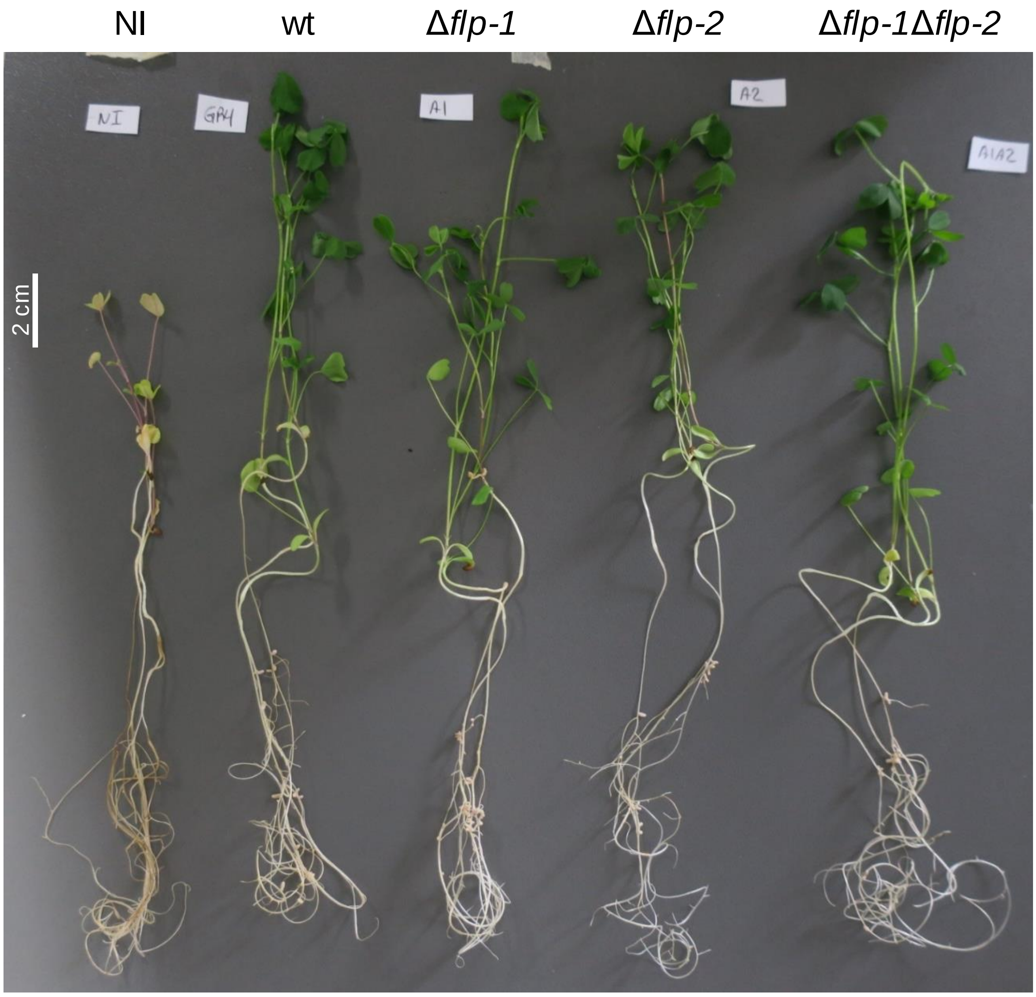

Figure S8. Appearance of alfalfa plants inoculated with *S. meliloti flp* mutant strains at the end of the nodulation kinetics experiment. Alfalfa plants were grown in hydroponic culture under axenic conditions in glass tubes. NI, non-inoculated; wt, wild-type strain GR4.

Table S1. Percent sequence identities (in parenthesis) among *S. meliloti* T4cP paralogues.

Locus GR4/Rm1021	Description	Chromosome *flp-1*	pSymA *flp-2*	pSymA Locus 3
**Chromosome *flp-1***				
C770_GR4Chr0141/*smc04118*	CpaK-like pseudopilin	*smc04117* (35% in 84 aa stretch)		
C770_GR4Chr0142/*smc04117*	CpaJ-like pseudopilin	*smc04118* (35% in 84 aa stretch)<br>smc04110 (*cpaD1*) (25% in 125 aa stretch)	*sma1572* (40% in 66 aa stretch)	
C770_GR4Chr0143/*smc04115*	CpaI-like putative pili related protein	*smc04111* (*cpaC1*) (21% in 103 aa stretch)	*sma1580* (36% in 97 aa stretch)	
C770_GR4Chr0144/*smc04114* (*pilA1*)	Flp pilin subunit	*smc02447* (59)	*sma1570* (*pilA2*) (60)	
C770_GR4Chr0145/*smc04113* (*cpaA1*)	Putative prepilin peptidase		*sma1578* (*cpaA2*) (37)	
C770_GR4Chr0146/*smc04112* (*cpaB1*)	Component of the outer membrane secretin complex		*sma1576* (*cpaB2*) (36)	
C770_GR4Chr0147/*smc04111* (*cpaC1*)	Component of the outer membrane secretin complex	*smc04115* (21% in 75 aa stretch)	*sma1580* (26% in 102 stretch)	*sma0163* (*pilQ2*) (30)
C770_GR4Chr0148/*smc04110* (*cpaD1*)	Component of the outer membrane secretin complex	*smc04117* (25)		
C770_GR4Chr0149/*smc04109* (*cpaE1*)	Putative docking protein of the secretion system		*sma1573* (*cpaE2*) (41)	
C770_GR4Chr0150/*smc02820* (*cpaF1*)	Pilus assembly ATPase		*sma1568* (*cpaF2*) (72)	
C770_GR4Chr0151/*smc02821*	CpaG-like putative inner membrane platform protein	*smc02822* (23)	*sma1564* (45)	
C770_GR4Chr0152/*smc02822*	CpaH-like putative inner membrane platform protein	*smc02821* (22)	*sma1562* (49)	
C770_GR4Chr0153/*smc04116* (TRm22)	Insertion sequence IS*Rm22*			
C770_GR4Chr0154/*smc02824*	CpaO-like TPR containing protein			
**Chromosomal orphan loci**				
C770_GR4Chr2732/*smc02446*	Flp pilin subunit	*smc04114* (*pilA1*) (59)	*sma1570* (*pilA2*) (64% in 25 aa stretch)	
C770_GR4Chr2733/*smc02447*	Hypothetical protein			
C770_GR4Chr3249/*smc03784*	CpaM-like polysaccharide deacetylase			
**pSymA *flp-2***				
C770_GR4pC0512/*sma1580*	CpaI-like putative pili related protein	*smc04115* (36% in 97 aa stretch)<br>*smc04111* (*cpaC1*) (26% in 102 aa stretch)		*sma0163* (*pilQ2*) (26% in 98 aa stretch)
C770_GR4pC0513/*sma1579*	Putative pili related protein			
C770_GR4pC0514/*sma1578* (*cpaA2*)	Putative prepilin peptidase	*smc04113* (*cpaA1*) (38)		
C770_GR4pC0515/*sma1576* (*cpaB2*)	Component of the outer membrane secretin complex	*smc04112* (*cpaB1*) (36%)		
C770_GR4pC0516/*sma1573* (*cpaE2*)	Putative docking protein of the secretion system	*smc04109* (*cpaE1*) (41)		
C770_GR4pC0517/*sma1572*	CpaJ-like pseudopilin protein	*smc04117* (40% in 66 aa stretch)		
C770_GR4pC0518/*sma1570* (*pilA2*)	Flp pilin subunit	*smc04114* (*pilA1*) (60)<br>*smc02447* (64% in 25 aa stretch)		
C770_GR4pC0519/*sma1568* (*cpaF2*)	Pilus assembly ATPase	*smc02820* (*cpaF1*) (72)		
C770_GR4pC0520/*sma1564*	CpaG-like putative inner membrane platform protein	*smc02821* (46)		
C770_GR4pC0521/*sma1562*	CpaH-like putative inner membrane platform protein	*smc02822* (49)		
**pSymA Locus 3**				
C770_GR4pC1351/*sma0163* (*pilQ2*)	RcpA-like putative component of the outer membrane secretin complex	*smc04111* (*cpaC1*) (30)	*sma1580* (26% in 98 aa stretch)	
C770_GR4pC1350/*sma0164*	Hypothetical lipoprotein			
C770_GR4pC1349/*sma0166*	CpaO/TadD-like TPR containing protein			

Table S2. Strains and plasmids used in this study.

Strain or plasmid	Relevant characteristics	Reference
Sinorhizobium meliloti		
GR4	Wild type, Nod$^+$, Fix$^+$	[57]
GR4flaAB	GR4 *flaAflaB*; Hyg^r	[10]
GRflp1	GR4 Δ*flp-1*	This work
GRflp2	GR4 Δ*flp-2*	This work
GRflp1flp2	GRflp1 Δ*flp-2*	This work
GRflaflp1	GRflp1 *flaAflaB*; Hyg^r	This work
GRflaflp2	GRflp2 *flaAflaB*; Hyg^r	This work
GRflaflp1flp2	GRflp1flp2 *flaAflaB*; Hyg^r	This work
Escherichia coli		
DH5*α*	*supE44, DlacU169, f80, lacZDM, 5hsdR171, recA1, endA1, gyrA96, thi-1, relA1*	Bethesda Research Lab
S17-1	*thi, pro, recA, hsdR, hsdM, RP4-2-Tc::Mu-Km::Tn7*	[82]
Plasmids		
pCR-XL-TOPO	Cloning vector; Km^r	Invitrogen
pTOPO-Δflp1	pCR-XL-TOPO carrying the deleted version of the *flp-1* cluster; Km^r	This work
pTOPO-Δflp2	pCR-XL-TOPO carrying the deleted version of the *flp-2* cluster; Km^r	This work
pK18*mobsacB*	Suicide plasmid; Km^r	[83]
pK18flaAB::Hyg	pK18*mobsacB* derivative carrying the mutation *flaAflaB*::Hyg; Km^r, Hyg^r	[10]
pK18-Δflp1	pK18*mobsacB* derivative carrying the deleted version of the *flp-1* cluster; Km^r	This work
pK18-Δflp2	pK18*mobsacB* derivative carrying the deleted version of the *flp-2* cluster; Km^r	This work
pGUS3	Plasmid containing *nfeD*::*gusA*, Km^r	[87]

Hyg^r and Km^r indicate hygromycin and kanamycin resistance, respectively.

Table S3. List of primers used in this study.

Name	Sequence (5′-3′)[a]	Use
Rev	CAGGAAACAGCTATGAC	Sequencing
Univ	GTTTTCCCAGTCACGAC	Sequencing
delpilA1.1	AAAGGATCCGTCTTAATTGCCGCTTGAGG (*Bam*HI)	Deletion in *flp-1*
delpilA1.2	GCTTGTCCTCGTTGCGTCCTTCATCAGG	
delpilA1.3	CCTGATGAAGGACGCAACGAGGACAAGC	
delpilA1.4	AAAGGATCCTCGAACGGACCATGACATCG (*Bam*HI)	
delpilA2.1	AAAAAGCTTCTGTTTCGGAGTCAGTCAGG (*Hin*dIII)	Deletion in *flp-2*
delpilA2.2	GTTCTCCTGTGCCAGCCGTATTCGATGG	
delpilA2.3	CCATCGAATACGGCTGGCACAGGAGAAC	
delpilA2.4	AAAAAGCTTTTCCTCTCAACGATCAAAGC (*Hin*dIII)	

[a]Restriction sites are underlined
